# Tracklet Pair Proposal and Context Reasoning for Video Scene Graph Generation

**DOI:** 10.3390/s21093164

**Published:** 2021-05-02

**Authors:** Gayoung Jung, Jonghun Lee, Incheol Kim

**Affiliations:** Department of Computer Science, Kyonggi University, Suwon-si 16227, Korea; jgyy4775@kyonggi.ac.kr (G.J.); jhlee17139@kyonggi.ac.kr (J.L.)

**Keywords:** video scene graph, visual relationship detection, tracklet pair proposal, spatio-temporal context reasoning, graph neural network

## Abstract

Video scene graph generation (ViDSGG), the creation of video scene graphs that helps in deeper and better visual scene understanding, is a challenging task. Segment-based and sliding-window based methods have been proposed to perform this task. However, they all have certain limitations. This study proposes a novel deep neural network model called VSGG-Net for video scene graph generation. The model uses a sliding window scheme to detect object tracklets of various lengths throughout the entire video. In particular, the proposed model presents a new tracklet pair proposal method that evaluates the relatedness of object tracklet pairs using a pretrained neural network and statistical information. To effectively utilize the spatio-temporal context, low-level visual context reasoning is performed using a spatio-temporal context graph and a graph neural network as well as high-level semantic context reasoning. To improve the detection performance for sparse relationships, the proposed model applies a class weighting technique that adjusts the weight of sparse relationships to a higher level. This study demonstrates the positive effect and high performance of the proposed model through experiments using the benchmark dataset VidOR and VidVRD.

## 1. Introduction

**Figure 1:**
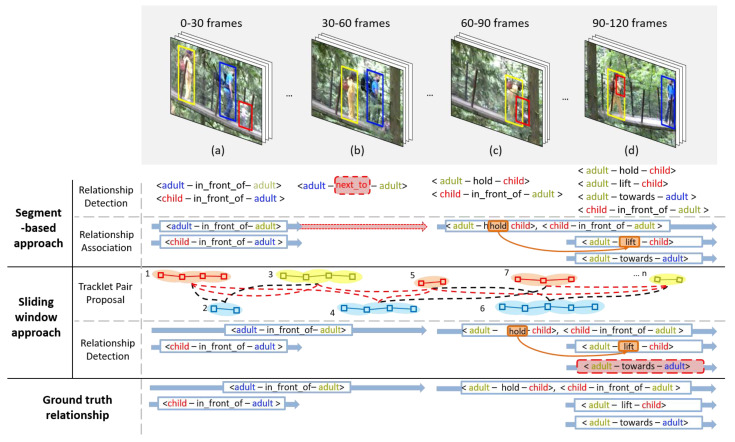
Different approaches to video scene graph generation.

Scene graphs are a graphical data structure in which objects appearing in a scene are represented by nodes and relationships between objects are represented by edges. These graphs are suitable representations for describing the scenes of an image or video. In various fields, such as visual question answering, semantic image retrieval, and image generation, scene graphs have proved to be a useful tool for deeper and better visual scene understanding [1]. Video scene graph generation (VidSGG) is the creation of multiple scene graphs that represent all objects in a video and the relationships between them. This task requires video relation detection (VidVRD) to find all the object tracklets in the video and the relationships between them, as shown in Figure 1. VidSGG is technically more challenging than generating a scene graph from a static image (ImgSGG) for two reasons [2]. The first reason is that spatio-temporal localization is needed instead of simple spatial localization for objects in VidSGG. In other words, it is necessary to find three-dimensional bounding boxes of various objects included in the video, while following the temporal axis. The second reason is that the relationships vary within the video. Unlike a single image, relationships of the same object pair may change over time and new relationships may also appear, making it difficult to predict relationships.

The design of a VidSGG model includes resolving a number of difficult issues. The first issue is how to detect object tracklets, which are three-dimensional bounding boxes of each object whose position and size change over time in a video. Previous studies [3,4,5,6,7] attempted a segment-based approach as shown in Figure 1. To avoid the complexity of finding object tracklets of different sizes and lengths in a long video and the relationship between them at once, the segment-based approach first divides the video into segments of constant length. The object tracklets and relationships between them in each segment were first searched, and then the same relationships detected in neighboring segments were connected to each other to integrate all of them into one relationship. For example, by integrating <adult-hold-child>, which is the same relationship independently detected in segments (c) and (d) in Figure 1, the relationship <adult-hold-child> is extended over the range of segments (c) to (d).

However, this segment-based approach has several limitations. Long-term relationships spanning multiple segments are repeatedly detected across such segments. For example, as shown in the upper part of Figure 1, relationships, such as <adult-hold-child> and <child-in_front_of-adult> must be duplicated in segments (c) and (d), requiring excessive computational cost. Moreover, if a relationship detection error or a relationship association error occurs in any of the intermediate segments in the relationship association process, when the relationships detected by dividing each segment are recombined, it is difficult to determine long-term relationships. For example, as shown in the upper part of Figure 1, failure to detect the relationship <adult-in_front_of-adult> in segment (b) prevents a long-lasting <adult-in_front_of-adult> from segments (a) to (b) from being discovered. Meanwhile, [8,9] presented a sliding window-based approach to solve the problems of the segment-based approach. This method detects object tracklets of different lengths and relationships between them by moving windows of various lengths on the video. Such a sliding window method is highly effective in detecting relatively long relationships, as shown in the lower part of Figure 1.

The second design issue of the VidSGG model is determining a tracklet pair proposal method that can effectively screen only those pairs that will actually have at least one relationship among the large number of object tracklet pairs detected in the video. Compared to the number of object pairs created in one image, the number of object tracklet pairs that can be created in a video consisting of a sequence of about 50 to 100 frames is much larger. Therefore, an effective tracklet pair proposal method is essential for efficient VidSGG. Such a method can have a greater impact on the efficiency of VidSGG for a sliding window-based model that generates multiple object tracklet pairs over the entire video range compared to segment-based models that generate a small number of object tracklet pairs within each segment. The object tracklets connected by dotted lines at the bottom of Figure 1 represent the number of object tracklet pairs that can be created by a sliding window-based model. [9] proposed a tracklet pair proposal method that evaluated tracklet pairs using spatial context and temporal context graphs. However, this method has a weakness in determining the relatedness of a corresponding tracklet pair only based on the temporal intersection over union (tIoU) and the spatial intersection over union (sIoU) between two object tracklets. For example, at the location of segment (d) in Figure 1, the relationship <adult-towards-adult> should be detected. However, as the distance between the two detected object tracklets is relatively long and do not spatially overlap each other, the model proposed by [9] assumes that the object tracklet pair has no relationship.

Meanwhile, the third design issue of the VidSGG model is the need to refine the features of the object and relationship nodes constituting the scene graph. In order to generate accurate video scene graphs, it is very important to extract and utilize various contexts about object tracklet pairs from a video. Therefore, it is necessary to determine which context is to be extracted and reflected in the object and relationship nodes. Owing to the nature of videos, it is important to utilize temporal as well as spatial context between object tracklets in order to create a video scene graph. In Figure 1 for example, the two relationships can be determined more accurately if the temporal context that the <adult-hold-child> relationship always appears in the video before the <adult-lift-child> relationship can be used. In previous studies, a fully-connected spatio-temporal graph was used to extract the spatio-temporal context between object tracklets; subsequently, the features of the object node of the graph were refined using this information [5,6]. This method helped refine the characteristic information of each node in the graph. However, these studies relied only on low-level visual context reasoning using only visual features of object tracklet pairs to extract and utilize contexts.

The final design issue of the VidSGG model is the limited classification accuracy of relationships that appear less frequently in the video. When classifying relationships using a machine learning model, such as a neural network, the classification accuracy tends to degrade for relationships that appear less often compared to relationships that appear often. This problem is called the long-tailed relationship distribution problem or relationship class imbalance problem. It is commonly encountered in VidSGG as well as other machine learning applications; solutions include over and under sampling, data augmentation, and class weighting.

To effectively cope with these various design issues, this study proposes a novel deep neural network model VSGG-Net for VidSGG. In order to overcome the limitations of the segment-based object tracklet detection method described earlier, the proposed model applies a sliding window scheme to detect object tracklets of various lengths. In particular, the proposed model uses a new tracklet pair proposal method that evaluates the relatedness of object tracklet pairs using a pretrained neural network and statistical information. Low-level visual context reasoning is performed using a spatio-temporal context graph and a graph neural network; high-level semantic context reasoning is also performed to effectively utilize the spatio-temporal context. To overcome the relationship class imbalance problem and improve the detection performance of sparse relationships, the proposed model applies a class weighting technique that raises the weight of sparse relationship in the classification loss function. This paper performs comparative experiments using two benchmark datasets, VidOR [10] and VidVRD [3], to analyze the effectiveness and performance of the proposed model, VSGG-Net, and presents the results. 

The contributions of this study can be summarized as follows.

- Important design issues for the VidSGG model are presented, and a novel deep neural network model, VSGG-Net, is proposed to effectively cope with these issues.
- A new tracklet pair proposal method that evaluates the relatedness of object tracklet pairs using the pretrained neural network and statistical information is presented.
- The proposed model performs low-level visual context reasoning and high-level semantic context reasoning using a spatio-temporal context graph and a graph neural network to obtain rich spatio-temporal context.
- The proposed model applies a class weighting technique that increases the weight of sparse relationships in the classification loss function to improve the detection performance for sparse relationships.
- The positive effect and high performance of the proposed model are proven through the experiments using the benchmark datasets, VidOR and VidVRD.

This study is organized as follows. Following the introduction in Section 1, Section 2 examines related studies, and Section 3 describes the design of the proposed VSGG-Net for video scene graph generation in detail. Section 4 introduces the model implementation and the experiments. Finally, Section 5 summarizes the conclusions.

## 2. Related Work

### 2.1. Visual Scene Graph Generation

Visual scene graph generation is a concept that has been studied for a long time to try to understand scenes by analyzing the objects contained in a single image and the relationships between them. Earlier computer vision tasks, such as object recognition, segmentation, and captioning have focused on coarser image understanding. Recently however, new tasks dealing with a finer level of image understanding have been actively introduced. Visual scene graph generation (SGG) is one such task; it expresses a single visual scene in a graphical structure [1]. A scene graph can convey detailed semantics of a video scene by explicitly modeling objects, their attributes, and relationships between them. In general, one of the most important parts in the SGG process is to refine the features of the graph using various contexts of objects.

The deep relational network proposed in [11] analyzed the spatial and statistical dependencies between two objects to determine their relationship. The spatial dependency between two objects was estimated by their proximity and relative spatial arrangement. Conversely, statistical dependency refers to the possibility of a specific relationship between two objects that can be statistically estimated. A bipartite graph with node GRU (Gated Recurrent Unit) and edge GRUs was constructed in [12] to obtain context between objects; an iterative message-passing scheme that transmitted messages between these GRU units was then presented.

In [13], a multi-level scene description network (MSDN) to refine features at different semantic levels, all while performing three tasks at the same time, was proposed considering the strong association between the three tasks: SGG, region captioning, and object detection. MSDN linked the object feature to the phrase feature, and the phrase feature to the caption feature, respectively, by exchanging information between these three semantic levels to update the features of each level at the same time. In [14], an attentional graph convolutional network (GCN) that allowed context to propagate across the graph along the edges of the graph was proposed. Using the attention of each edge, propagation was controlled to prevent information with little correlation between the two nodes from flowing to the edges. Conversely, MotifNet [15] broke away from the traditional paradigm of propagating information in both directions between objects and relationships. The model, instead, enabled the global contexts of all previous stages to facilitate prediction of subsequent stages by sequentially staging the bounding box prediction, object classification, and relationship classification.

### 2.2. Video Visual Relation Detection

Video scene graphs can be easily created using <subject, relationship, object> triplets, which are the results of video relationship detection. Hence, most of the existing studies have focused on video relation detection (VidVRD) rather than video scene graph generation (VidSGG). VidVRD is a task that detects object tracklets and relationships between them in a video composed of a sequence of multiple frames, not a single image. In [3], a new VidVRD task and a benchmark dataset, ImageNet-VidVRD v1.0, were introduced for the first time. [3] also divided the video into fixed-length segments, detected relationships between object tracklets in each segment, and presented a baseline model combining the same relationships detected in each segment together.

In VidVRD and VidSGG, it is important to find object tracklets having various temporal and spatial sizes in the video. Existing models in [4,5,6,7] divided the video into fixed-length segments and detected the object tracklets and relationships between them, like the baseline model in [3]. However, as described earlier, this segment-based approach has problems such as redundant detection of the same relationship in multiple segments and difficulty in finding long-term relationships when an error occurs in relationship detection or relationship association for each segment. In [5,7], additional improved relationship association methods were proposed to complement the limitations of this segment-based approach. In the model of [7], the relationship association method using the Siamese neural network was presented. In the model of [7], the multiple hypothesis association (MHA) method was presented. Meanwhile, in [8,9], unlike a segment-based approach that divides video into segments, a sliding window scheme was attempted. The model of [8] detects object tracklets over the entire video range through object detection and object tracking. It then determines the relationships between these pairs. In addition, a sliding window was used in the post-processing process to accurately readjust the length of the detected relationships. By contrast, the model in [9] applied a sliding window technique to the process of detecting object tracklets in video before determining relationships. In order to suppress redundant detection of the same object tracklets, track-level non-maximum suppression (NMS) was applied in the model of [8], and object-level NMS was used in the model of [9]. This sliding window technique resolves the limitation of the segment-based approach, but causes another problem by increasing the number of object tracklet pairs to determine a relationship due to the large number of object tracklets found in the entire video range. Therefore, a tracklet pair proposal for sliding window-based models was required, which only selects tracklet pairs that actually have a relationship. In [9], a tracklet pair proposal method, which extracts spatio-temporal context between object tracklets using spatial GCN and temporal GCN and evaluates each object tracklet pair using this information, was presented. Different from [9], our proposed model uses a novel tracklet pair proposal method where the relatedness of a tracklet pair is measured based upon both the pretrained neural network and the statistical information.

To accurately determine the relationship type between two object tracklets in a video, it is important to utilize various features and contexts about the two object tracklets. In the model of [4], the spatial–temporal and language context features of the object tracklets were used in addition to the visual features mainly used in previous studies. The spatio-temporal feature was composed of the relative location of two object tracklets and the motion of each object tracklet. The language context feature was created as the concatenation of subject and object category embedding vectors. Meanwhile, in the models of [5,6], a fully-connected spatio-temporal graph was used to extract the spatio-temporal contexts between object tracklets. The features of the object node of the graph were refined based on the context. Whereas the model of [6] built a spatio-temporal graph only with object tracklets within the same segment, the model of [5] constructed a spatio-temporal graph including object tracklets within the same segment as well as in adjacent segments. In addition, the model in [6] used the conditional random field (CRF), a probabilistic graphical model, and the model in [6] used the GCN, a neural network for context reasoning. However, both models only performed low-level visual context reasoning using the visual features of object tracklets. They did not perform the high-level semantic reasoning proposed in this study. Different from models [5,6], the proposed model VSGG-Net performs both low-level visual context reasoning and high-level semantic context reasoning based on two-level spatio-temporal context graphs to obtain rich spatio-temporal context features.

Like in ImgSGG, long-tailed class distribution or class imbalance problems occur in VidVRD and VidSGG. In other words, classification accuracy is low for the objects or relationships of a class that appear less frequently in video, compared to the objects or relationships of classes that appear frequently. In [8], extra training data for sparse object classes were obtained from the Microsoft COCO dataset [16] to overcome the object class imbalance problem, and the conventional softmax loss was replaced with the focal loss. Unlike [8], our proposed model uses a class weighting method to solve the relationship class imbalance problem.

## 3. VSGG-Net: The Video Scene Graph Generation Model

### 3.1. Model Overview

**Figure 2:**
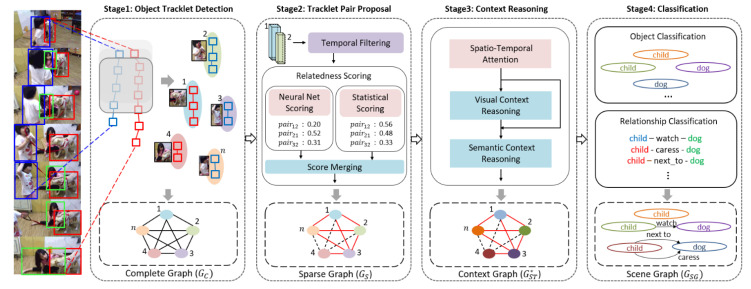
The proposed model for video scene graph generation.

This paper proposes a new deep neural network model, VSGG-Net, for video scene graph generation (VidSGG). VSGG-Net includes: object tracklet detection to find spatio-temporal regions of objects of various sizes over the entire video range using a sliding window scheme; a tracklet pair proposal to select only those with high relatedness among object tracklet pairs appearing in the video; hierarchical context reasoning based on spatio-temporal graph neural network to extract rich context between two objects; and object and relationship classifications applying class weighting technique to solve the long-tailed relationship distribution problem. The structure of the proposed model is shown in Figure 2. The proposed model can be viewed as a single pipeline consisting of four stages including object tracklet detection (OTD), tracklet pair proposal (TPP), context reasoning (CR), and object and relation classification (ORC).

In the OTD stage of the proposed model, the video is not divided into segments of a fixed length. Instead, windows of different sizes are moved on the video using a sliding-window technique; object tracklets of different duration are detected over the entire video range. A complete graph, $G_{c}$, was created by connecting the nodes representing these object tracklets. $G_{c}$ is a graph created by assuming that at least one binary relationship exists between all pairs of objects in a video. However, as the number of objects appearing in the video, n, increases, _n_C_2_ = n(n − 1)/2, which is the number of all possible object pairs that can be related, increases exponentially. Therefore, a tracklet proposal that selects only tracklet pairs of objects most likely to have a relationship is important for an efficient VidSGG. In the TPP stage of the proposed model, the relatedness of each object tracklet pair is evaluated by combining the pretrained neural net scoring and statistical scoring based on the data set. Then, only pairs of object tracklets whose relatedness is higher than a certain level are selected to generate a sparse graph $G_{s}$. In general, it is important to utilize various spatial and temporal contexts to determine a specific relationship between two objects appearing in a video. In the CR stage of the proposed model, a spatio-temporal contextualized graph, $G_{ST}^{*}$, containing abundant spatial and temporal contexts between object tracklets is derived through a hierarchical reasoning process using a spatio-temporal graph neural network. Finally, the feature representations of each object node and each relationship node of the spatio-temporal contextualized graph are used in the ORC stage of the proposed model. The final video scene graph $G_{SG}$ is generated by determining the object class and relationship type corresponding to the node. The notations used in this paper are summarized in Table 1.

### 3.2. Object Tracklet Detection and Pair Proposal

In the OTD stage of the proposed model, VSGG-Net, an object detector, is used to find two-dimensional (2D) spatial regions of objects in each video frame. Object tracklets, which are the spatio-temporal regions, in which each object appears in the video, must be detected based on these. A sliding window scheme [9] that moves multiple windows of different sizes across the entire video range is used to effectively detect object tracklets of various lengths instead of dividing the video into segments of a fixed size. In the proposed model, a Faster-RCNN [17] with ResNet101 [18] backbone is used as an object detector for object detection frame by frame. This object detector is used after training with datasets of MS-COCO [16] and ILSVRC2016 [19]. After object detection is performed for each frame, the same object is connected between neighboring frames over the entire range of the video to find object tracklets. The proposed model uses the Deep Sort [20] algorithm for such object tracking. After the basic object tracklets are detected over the entire range of the video, a sliding window technique is applied to find object tracklets of various lengths based on these basic object tracklets. In order to find object tracklets of various lengths, windows of various sizes are set and used starting with a minimum size of 30 frames. After detecting object tracklets of various lengths over the entire range of the video using the sliding window technique, a complete graph, $G_{c}$, is generated, assuming that at least one relationship exists between all pairs of detected object tracklets. Each node of this graph $G_{c}$ represents one object tracklet and each edge represents the relationship between the two objects.

**Figure 3:**
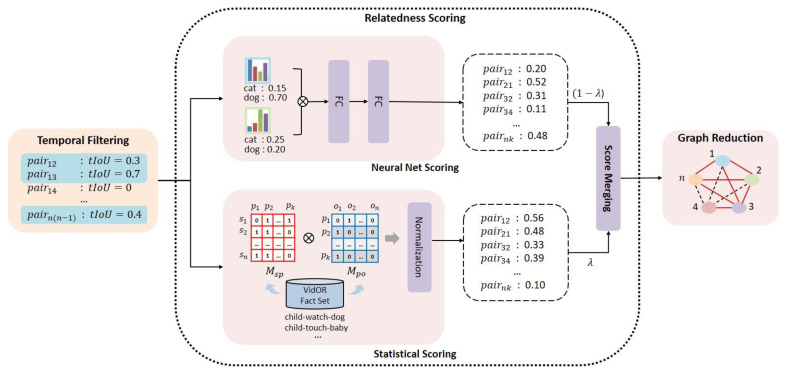
Tracklet pair proposal (TPP).

As the number n of object tracklets detected in the entire video range, not in each segment range, is very large, _n_C_2_ = n(n − 1)/2, which is the number of all possible object tracklet pairs, places a heavy burden on the overall VidSGG task. Therefore, a task is performed in the object tracklet pair proposal (TPP) stage of the proposed model to select only pairs of object tracks having a high relationship after evaluating the relatedness of each pair of object tracklets. The relatedness scoring for each pair of object tracklets is performed by combining the trained neural network-based evaluation and the dataset-based statistical scoring. Through this process, edges with low relatedness are excluded in the TPP stage from the complete graph $G_{c}$ obtained in the OTD stage, and a sparse graph $G_{s}$ that is more compact is generated. The mechanism of object tracklet pair proposal of the proposed model, VSGG-Net, is schematically shown in Figure 3.

Prior to evaluating the relationship between each object pair, temporal filtering (TF) is performed using tIoU, which represents the temporal overlapping between two object tracklets. In the video, object tracklet pairs that do not overlap at all in time, such as $tIoU\left(o_{i},\;o_{j}\right)=0$, are excluded from the set of candidate object tracklet pairs on the assumption that they cannot have any relationship. For object tracklet pairs that have passed temporal filtering, the relatedness between the two object tracklet pairs is evaluated. To evaluate the relatedness between object tracks, neural net scoring and statistical scoring are used together, as shown in Figure 3. Neural net scoring uses a neural network that determines the suitability of the object tracklet pair based on the class distribution of each of the two object tracklets. For example, when there are three classes of objects: “cat”, “plate”, and “vegetable”, the suitability of the pair (“vegetable”, “plate”) is determined to be higher than that of (“cat”, “plate”) by the pretrained neural network. The neural network used for relatedness scoring is composed of two fully-connected layers, and is used after pretraining with the VidOR training dataset. If at least one relationship exists between two object tracklets on the scene graph of the VidOR training dataset, the corresponding object tracklet pair is regarded as a positive example for neural network training. Otherwise, it is regarded as a negative example. Therefore, the relatedness score for the object tracklet pair $\left(o_{i},\;o_{j}\right)$ using a neural network is calculated as Equation (1).

(1) $$score_{NN}\left(o_{i},\;o_{j}\right)=\left[FC_{1}\left(p_{i}\right)\;\cdot FC_{2}\left(p_{j}\right)\right],i\;\neq \;j\;$$

In Equation (1), $p_{k}$ represents a class distribution map of each object forming an object tracklet pair and $FC_{m}\left(p_{k}\right)$ describes a fully connected layer.

Another method of evaluating the relatedness between two object tracklets is statistical scoring. Each scene graph included in the VidOR training dataset can be viewed as a set of facts in the form of a triplet, such as <subject, relationship, object>. Statistical scoring evaluates the relationship between two objects according to the frequency indicating how often two objects co-occur as a subject and an object in this set of facts. For statistical scoring, each fact is divided into (subject, relation_predicate), and (relation_predicate, object) to create two co-occurrence matrices, $M_{sp}$ and $M_{po}$, as shown in Figure 3. The matrix $M_{so}$, containing the relational scores for all possible (subject, object) pairs, is calculated by multiplying these two matrices. By normalizing the scores in $M_{so}$ for each object tracklet pair $\left(o_{i},\;o_{j}\right),$ the statistical relatedness score, $score_{ST}\left(o_{i},\;o_{j}\right)\;$is obtained.

For each object tracklet pair, $\left(o_{i},\;o_{j}\right)$, the final relatedness score, $score_{MIX}\left(o_{i},\;o_{j}\right)\;$, is calculated by combining the neural net score $score_{NN}\left(o_{i},\;o_{j}\right)\;$and the statistical score $score_{ST}\left(o_{i},\;o_{j}\right),\;$as shown in Equation (2).

(2) $$score_{MIX}\left(o_{i},\;o_{j}\right)=\left(1-\lambda \right)\cdot score_{NN}\left(o_{i},\;o_{j}\right)+\lambda \cdot score_{ST}\left(o_{i},\;o_{j}\right)\;$$

λ in Equation (2) means the weight can adjust the relative reflection ratio of neural net score $score_{NN}\left(o_{i},\;o_{j}\right)\;$and the statistical score $score_{ST}\left(o_{i},\;o_{j}\right)\;$according to the reliability of the two scoring methods. For the VidOR dataset, λ is set to 0.7 in the proposed model. Only object tracklet pairs with a total relatedness score $score_{MIX}\left(o_{i},\;o_{j}\right)\;$higher than the threshold value (threshold) of 0.7 or higher than $score\left(o_{j},\;o_{j}\right)\;(\geq \;0.7$) are proposed in this stage. In the TPP stage, all edges connecting two object tracklet nodes with no relationship or very low relationship are excluded from the complete graph $G_{c}$ to generate the sparse graph $G_{s}$.

### 3.3. Context Reasoning and Classification

**Figure 4:**
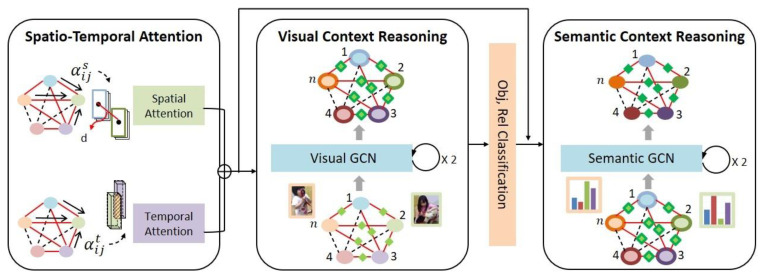
Context reasoning (CR).

In order to effectively discriminate between various objects appearing in the video and their relationship, temporal contexts are needed in addition to various spatial contexts. In the CR stage of VSGG-Net, a CR based on a spatio-temporal graph neural network is applied to the sparse graph $G_{s}$ generated by TPP to generate a context graph $G_{ST}^{*}$ containing rich spatio-temporal contexts. Figure 4 shows the CR process of the proposed model.

As shown in Figure 4, the context reasoning process of VSGG-Net is largely composed of the following stages: spatio-temporal attention, visual context reasoning, and semantic context reasoning. Visual context reasoning uses the visual information of each object tracklet, whereas semantic context reasoning uses the class distribution value of each object tracklet, which is the result of visual context reasoning. Therefore, the CR process of the proposed model is a hierarchical reasoning process consisting of lower-level visual context reasoning and higher-level semantic context reasoning. In addition, the CR of each level is performed iteratively using pre-calculated spatial attention $\alpha_{ij}^{S}$, temporal attention $\alpha_{ij}^{T}$, and the GCN [21]. The GCN repeats the process of updating to include sufficient spatio-temporal contexts in each node by reflecting information of neighboring nodes to each node based on the temporal attention and the spatial attention. In the proposed model, a context graph is first constructed to perform CR. The context graph has two types of nodes, an object node, and a relationship node. It also has three types of edges that connect the pairs of (subject node, relationship node), (relationship node, object node), and (subject node, object node), respectively. Unlike the existing GCN [21], the spatio-temporal GCN of the proposed model not only uses the spatial attention $\alpha_{ij}^{S}$ and the temporal attention $\alpha_{ij}^{T}$, but also enables exchanging information between the node pairs (subject node, relationship node) and (relationship node, object node) as well as (subject node, object node) through the three types of edges.

The detailed CR process of each level is as follows. First, each subject and object node of the initial visual context graph for visual context reasoning are filled with I3D visual features of the object tracklets and CNN visual features of frames belonging to the tracklet range. Each relationship node is filled with I3D visual features of each subject tracklet and object tracklet that have corresponding relationship and relative features [1] of the (subject, object) pair. Equation (3) represents the three types of relative features used in the proposed model. In Equation (3), $C_{s}^{i}$ and $C_{o}^{i}$ denote the center coordinates of the bounding box of the subject tracklet and the object tracklet, respectively; $S_{s}^{i}$ and $S_{o}^{i}$ denote the size of the bounding box of the subject tracklet and the object tracklet, respectively.

(3) $$\Delta C=\left(C_{s}^{1}-C_{o}^{1},\ldots ,C_{s}^{L}-C_{o}^{L}\right),\;\Delta C=\left(C_{s}^{1}-C_{o}^{1},\ldots ,C_{s}^{L}-C_{o}^{L}\right),\;\Delta M=\left(\Delta C^{2}-\Delta C^{1},\ldots ,\Delta C^{L}-\Delta C^{L-1}\right)$$

When the initial visual context graph is created, the spatial attention $\alpha_{ij}^{S}$ and the temporal attention $\alpha_{ij}^{T}\;$to be applied to each edge connecting two object nodes are calculated. As two object tracklets $o_{i}$ and $o_{j}$ are located closer in space, the spatial attention $\alpha_{ij}^{S}$ on the edge between the corresponding object nodes should be strengthened. Furthermore, as two object tracklets $o_{i}$ and $o_{j}$ are overlapped longer in time, the temporal attention $\alpha_{ij}^{T}\;$on the edge between the corresponding object nodes should be also strengthened. Therefore, the spatial attention $\alpha_{ij}^{S}$ and the temporal attention $\alpha_{ij}^{T}\;$are computed using Equations (4) and (5), respectively.

(4) $$\alpha_{ij}^{S}=max\left(d\left(o_{i},\;o_{N_{i}}\right)\right)\;/\;\sum_{j\;\in \;N_{i}^{s}}\left(d\left(o_{i},\;o_{j}\right)\right)$$

(5) $$\alpha_{ij}^{T}=exp\left(tIoU\left(o_{i},\;o_{j}\right)\right)\;/\;\sum_{j\;\in \;N_{i}^{T}}\left(exp\left(tIoU\left(o_{i},\;o_{j}\right)\right)\right)$$

In Equation (4), $d\left(o_{i},\;o_{j}\right)$ denotes the distance between the centroids of the two object tracklets, and $N_{i}^{S}$ denotes a set of other object tracklets spatially adjacent to the subject tracklet. In Equation (5), $tIoU\left(o_{i},\;o_{j}\right)$ denotes the degree of temporal overlap between two object tracklets, and $N_{i}^{T}$ denotes a set of other object tracklets temporally adjacent to the subject tracklet. Using the pre-calculated spatial attention $\alpha_{ij}^{S}$ and temporal attention $\alpha_{ij}^{T}\;$, each subject node and object node in the spatio-temporal context graph is updated, as shown in Equation (6). Each relationship node is updated, as shown in Equation (7), reflecting the contexts of the neighboring nodes.

(6) $$z_{i}^{o}=\sigma \left(\alpha W_{so}Z^{o}+W_{sr}Z^{r}+W_{ro}Z^{r}\right)$$

(7) $$z_{i}^{r}=\sigma \left(z_{i}^{r}+W_{rs}Z^{o}+W_{ro}Z^{o}\right)$$

In Equations (6) and (7), s, r, and o denote a subject node, a relationship node, and an object node, respectively. Attention α denotes the sum of spatial attention and temporal attention ($(\alpha^{S}+\alpha^{T}$). In Equations (6) and (7), $Z^{o}$ denotes information received from neighboring subject and object nodes, and $Z^{r}$ denotes information received from neighboring relation nodes. In Equations (6) and (7), $W_{so}$, $W_{sr}$, $W_{ro}$, and $W_{rs}$ denote weights between the subject–object, subject–relationship, relationship–object, and relationship–subject nodes, respectively. As expressed in Equations (6) and (7), visual context reasoning is performed while passing through the two spatio-temporal GCN layers.

When the visual context reasoning is completed, each object node in the visual context graph uses an object classifier and each relationship node uses a relationship classifier to calculate the class distribution to which the corresponding node belongs. In order to start performing semantic context reasoning, a new semantic context graph with the same structure as the visual context graph is generated. Each node of this semantic context graph is initialized with the probability distribution for each class of the visual context graph node corresponding to this node. Semantic context reasoning is performed through the two spatio-temporal GCN layers in the same way as visual context reasoning. In this case, the same spatial attention $\alpha_{ij}^{S}$ and temporal attention $\alpha_{ij}^{T}$ as for visual context reasoning are used. The final spatio-temporal context graph $G_{ST}^{*}$ obtained through this process has a higher-level semantic context based on a lower-level visual context.

**Figure 5:**
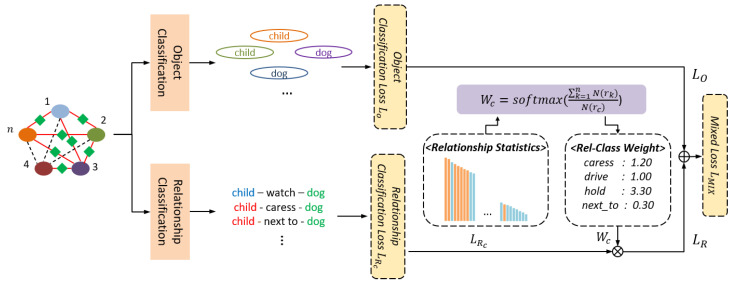
Object and relationship classification (ORC).

In the ORC stage of VSGG-Net, the objects constituting the video scene graph and the relationships between them are determined using the spatio-temporal context graph $G_{ST}^{*}$. In this stage, the object nodes and relational nodes are classified into the most likely categories, based on the information of each node in the spatio-temporal context graph $G_{ST}^{*}$. Figure 5 shows the process of classifying objects and relationships in the proposed model. Each object node in the spatio-temporal context graph $G_{ST}^{*}$ passes through a softmax function and labeled with the object class of the highest score. Each relationship node passes through a softmax function and labeled with the top five relationship classes of the highest scores. For object classification, cross entropy is used as the loss function; for relationship classification, binary cross entropy is used as the loss function. This allows the proposed model to have various relationships for one object pair at the same time. In other words, the proposed model allows an object pair of (“child”, “dog”) to have multiple relationships such as child-caress-dog and child-next_to-dog at the same time as shown in Figure 5.

In datasets such as VidOR and VidVRD, there are relationships that appear with high frequency, such as “next_to” and “in_front_of”, as well as many relationships appearing at a low frequency, such as “cut” and “shake_hand_with." Relationships with a low frequency of appearance inevitably have a lower recognition rate compared to those with a high frequency of appearance in the relationship classification process. In the ORC stage of VSGG-Net, a relationship class weighting technique, as shown in Equation (8), is applied to solve the long-tailed relationship distribution problem. This technique adjusts the weight of the loss of the relationship class in the loss function according to the frequency of appearance. According to this technique, the lower the frequency of appearance, the higher the weight of the relationship loss in the loss function, as shown in Equation (8). In Equation (8), $N\left(r_{k}\right)$ denotes the number of relationship instances corresponding to the relationship class k in the training dataset, and $w_{c}$ denotes the weight of the relationship class c in the loss function $L_{R}$ for learning the relationship classifier.

(8) $$w_{c}=softmax\left(\frac{\sum_{k=1}^{n}N\left(r_{k}\right)}{N\left(r_{c}\right)}\right)$$

By applying this relationship class weighting technique, VSGG-Net can obtain a high classification performance even for relationships with a relatively low frequency of appearance.

## 4. Experiments

### 4.1. Dataset and Model Training

A performance evaluation experiment of VSGG-Net is performed using two benchmark datasets, VidOR [10] (https://xdshang.github.io/docs/vidor.html) and VidVRD [3] (https://xdshang.github.io/docs/imagenet-vidvrd.html). The VidOR video dataset includes 80 object types and 50 relationship types. The relationships in VidOR are largely divided into spatial relationships, such as “above” and “behind” and action relationships, such as “drive” and “chase.” The VidOR dataset, consisting of a total of 10,000 videos, is divided into 7000 training data, 835 validation data, and 2165 test data. Meanwhile, the VidVRD video dataset includes 35 object types and 132 relationship types. The VidVRD dataset, consisting of a total of 1000 videos, is divided into 800 training data and 200 test data.

The proposed model, VSGG-Net, is implemented using PyTorch, a Python deep learning library, in an Ubuntu 16.04 LTS environment. The model is trained and evaluated in a hardware environment with a GeForce GTX 1080Ti GPU card installed. For model training, the batch size is set to 4 and the epoch is set to 10. In addition, the learning rate is 0.001, and the stochastic gradient descent (SGD) is used as the optimizer. The tracklet pair proposal subnet of the proposed model has a total of 19,760 trainable weights, while the context reasoning and classification subnet includes a total of 63,872,665 trainable weights.

### 4.2. Experiments

The first experiment is conducted to prove the positive effect of the tracklet pair proposal (TPP) used in VSGG-Net. This experiment compares the following cases: no separate tracklet pair proposal mechanism is applied (None); only temporal filtering is applied (TF); temporal filtering and neural net-based relatedness scoring are applied (TF+NS); temporal filtering and statistical relatedness scoring are applied (TF+SS); and temporal filtering, neural net-based relatedness scoring, and statistical relatedness scoring are all applied as in VSGG-Net (TF+NS+SS). The number of the proposed tracklets, as well as the recall R@1 and the precision P@5 of the object tracklet pairs, are used as performance metrics. The performance metrics used in experiments are listed in Table 2. An average of 986 object tracklets appear in the video of the VidOR dataset used in this experiment.

Table 3 shows the results of this experiment. TF+NS+SS, like VSGG-Net, and TF+SS showed the highest performance in terms of recall R@1 and precision P@5. It was followed by TF+NS, TF, and None in terms of precision P@5. This result suggests that temporal filtering and two relatedness scoring methods used in the proposed model are very effective for tracklet pair proposal. In addition, statistical relatedness scoring is relatively more effective than neural net-based scoring in general. Meanwhile, in terms of the number of proposed object tracklets, TF+NS+SS and TF+SS significantly reduced the number of object tracklets compared to None or TF, but increased the object tracklets by a slightly larger number compared to TF+NS. Considering that both TF+NS+SS and TF+SS had higher recall and precision than TF+NS, the performance of TF+NS+SS and TF+SS for tracklet pair proposal is relatively higher. Based on these experimental results, the temporal filtering and the two relatedness scoring methods proposed in this paper are effective in the object tracklet pair proposal, and the effect is maximized when they were used together.

The second experiment is conducted to prove the superiority of the CR method of VSGG-Net using a spatio-temporal graph neural network. This experiment compares the following cases: no separate attention is applied (GCN); only spatial attention is applied (S-GCN); only temporal attention is applied (T-GCN); and both spatial attention and temporal attention are applied as in VSGG-Net (ST-GCN). In addition, the relation detection performance and relation tagging performance are measured for each of the four cases. The relation detection task takes a video as input to output a set of relation triplets with localized objects. A relation triplet is considered to be correct if the same relation triplet is tagged in the ground truth and both trajectories of its subject and object have sufficient vIoU (volume Intersection over Union). The relation tagging task reduces the influence of object localization, the output of which is a set of video relation triplets annotated to the whole video without the localization of the object [4,8].

Table 4 shows the results of this experiment. ST-GNN applying both spatial attention and temporal attention, as in VSGG-Net, demonstrated the highest performance of all cases. Conversely, GCN applying neither spatial attention nor temporal attention demonstrated the lowest performance as no spatio-temporal contexts were used. In addition, T-GCN applying only the temporal attention showed slightly higher performance than S-GNN applying only the spatial attention. These results confirm once again that the use of temporal context is important in the video scene graph generation task. The experimental results suggest that the CR method of VSGG-Net using a spatio-temporal graph neural network is highly effective at improving the VSGG performance.

The third experiment is conducted to compare the performance of VSGG-Net according to the level of context reasoning. This experiment compares the cases of only visual reasoning, only semantic reasoning, and visual reasoning + semantic reasoning as in the proposed model. In addition, the relation detection performance and relation tagging performance are measured for each of the three cases.

Table 5 shows the results of this experiment. The case of performing both visual reasoning and semantic reasoning, which were context reasoning methods of different levels as in VSGG-Net, demonstrated the highest performance of all cases. In addition, the case performing only visual reasoning showed higher performance than the case performing only semantic reasoning. These results suggest that visual reasoning using visual information is important in the video scene graph generation task. Using both context reasoning methods, as in VSGG-Net, also proved to be effective in detecting various types of relationships appearing in the video.

The fourth experiment is conducted to confirm the superiority of VSGG-Net by comparing the performance with existing models. Of the compared models, MAGUS.Gamma [4] and MHA [7] are segment-based models that divide videos into segments, whereas RELAbuilder [8], VRD-STGC [9], and VSGG-Net are sliding window-based models. The RELAbuilder [8] model uses the sliding window during post-processing to accurately readjust the length of the detected relationships. The VRD-STGC [9] model and VSGG-Net use the sliding window when detecting object tracklets prior to relationship prediction.

Table 6 shows the experimental results using the validation set of VidOR. VSGG-Net shows better performance than the existing models in both relation detection and relation tagging. Compared to the segment-based model MAGUS.Gamma [4], the proposed model demonstrates an improved performance of 18.28% in R@50, 30.57% in R@100, and 49.39% in mAP (mean average precision), which are performance measures for relation detection. It also demonstrated an improved performance of 14.14% in P@1 and 32.97% in P@5, which are performance measures for relation tagging. Compared to the VRD-STGC [9] model using a similar sliding window technique, the proposed model demonstrates an improved performance of 16.46% in R@100 and 43.06% in mAP, which are performance measures for relation detection. It also demonstrated an improved performance of 19.46% in P@1 and 47.25% in P@5, which are performance measures for relation tagging. The proposed model shows the most remarkable performance improvement in mAP of relation detection and both P@1 and P@5 of relation tagging. These experimental results demonstrate the outstanding performance improvement of the context reasoning method of VSGG-Net using a spatio-temporal context graph and a graph neural network.

Meanwhile, another sliding window-based model, VRD-STGC [9], shows higher performance than the segment-based models MAGUS.Gamma [4] and MHA [7], which could be interpreted as confirming the superiority of sliding window-based models compared to segment-based models. However, RELAbuilder [8], a model that uses a sliding window for post-processing after relationship detection, shows the lowest performance of the compared models, suggesting that the sliding window technique during post-processing had little effect on improving performance.

Meanwhile, Table 7 shows the experimental results using the test set of VidVRD, another benchmark dataset. In this experiment, new models (VidVRD [3], VRD-GCN [5], and GSTEG [6]) are also compared. VSGG-Net shows better performance than the existing models in both relation detection and relation tagging. The proposed model demonstrates an improved performance of 8.86% in R@50, 44.17% in R@100, and 6.71%, 7.27%, and 13.68% in P@1, P@5, and P@10, respectively, compared to the MHA [7] model, which has the highest performance of the segment-based models. The proposed model also demonstrates an improved performance of 0.8% in R@50, 0.36% in R@100, and 2.26%, 3.03%, and 3.84% in P@1, P@5, and P@10, respectively, compared to the VRD-STGC [9] model, which is a sliding window-based model. However, when using the test set of VidVRD, the MHA [7] model shows the highest performance in mAP. Table 5 and Table 6 show experimental results using different datasets, VidOR and VidVRD, confirming the generality and scalability of VSGG-Net.

### 4.3. Qualitative Analysis

**Figure 6:**
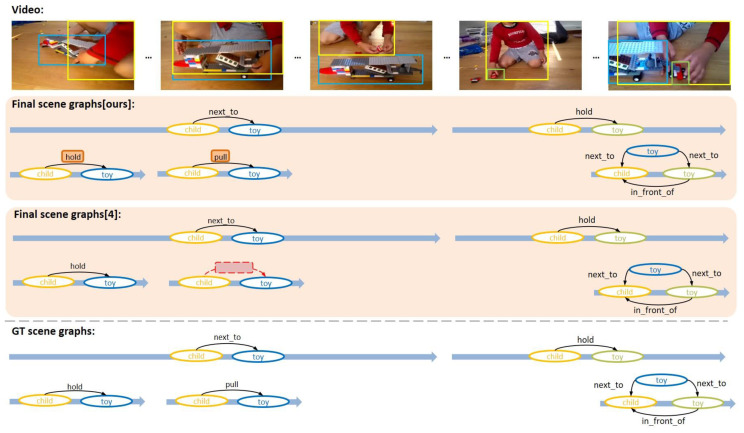
First example of VidSGG task performed by the proposed model.

**Figure 7:**
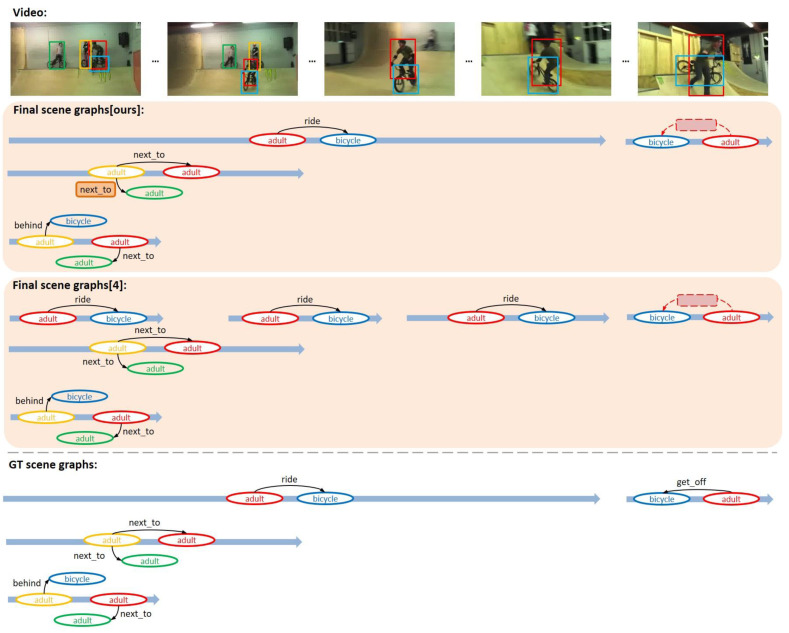
Second example of VidSGG task performed by the proposed model.

**Figure 8:**
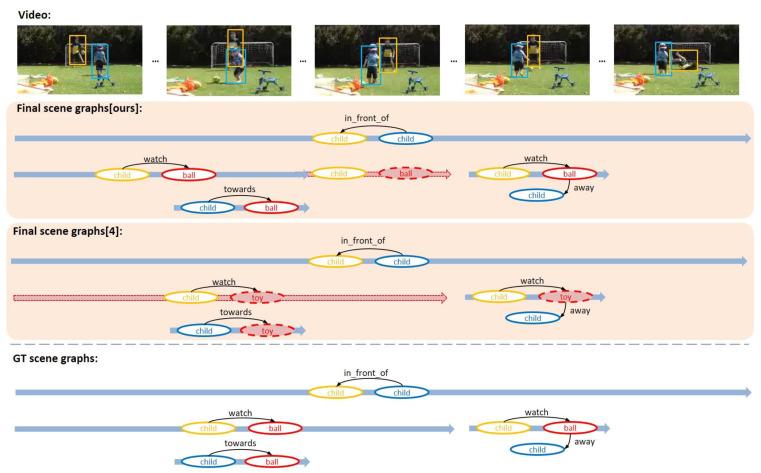
Third example of VidSGG task performed by the proposed model.

In order to qualitatively evaluate the performance of VSGG-Net, some examples in which the proposed model generated a scene graph among VidOR benchmark data are used. In Figure 6, Figure 7 and Figure 8, the upper part of each image shows the input video, the middle part shows the final generated scene graphs, and the lower part shows the ground truth scene graphs.

Figure 6 is an example of VSGG-Net generating correct scene graphs from the input video to match the ground truth scene graphs. Only two objects appeared in this scene, “child” and “toy.” All relationships detected by VSGG-Net, such as <child-next_to-toy>, <child-hold-toy>, <child-pull-toy>, <child-hold-toy>, <child-in_front_of-toy>, <toy-next_to-child>, and <toy-next_to-toy>, match with the ground truths not only in terms of the triplets but also in terms of the temporal ranges of the relationship. Such results show the relatively high object tracklet detection performance and tracklet pair proposal performance of the proposed model. In addition, the proposed model predicted the two action relationships <child-hold-toy> and <child-pull-toy> in order by effectively utilizing temporal context information by context reasoning. However, the existing MAGUS.Gamma model [4] did not detect the relationship <child-pull-toy> following <child-hold-toy>, because it could not make use of temporal context information. The results show the power of our VSGG-Net’s context reasoning capability.

In Figure 7, as in Figure 6, the objects and relationships detected by the proposed model and the scene graphs generally match the ground truths. In particular, it succeeded in detecting the “next_to” relationship even for two “adult” object tracklets that are separated by some distance. Another sliding window-based model, VRD-STGC [9], determined that these two “adult” object tracklets that did not spatially overlap each other did not have a relationship with each other in the tracklet pair proposal stage. Meanwhile, the proposed model failed to detect the relationship <adult-get_off-bicycle> appearing at the end of the video. This relationship appeared and disappeared very briefly in the video, occurring in approximately 10 frames. Because the minimum length of sliding window used by the proposed model is set to 30 frames, it may have been difficult to detect object tracklets with shorter lengths and the relationship between them. In order to detect exceptionally short or long relationships like this, hyperparameters of the model should be set to allow sliding windows of more various sizes to be used. We notice the segment-based MAGUS.Gamma model [4] detected only multiple segmented relationships <adult-ride-bicycle> without finding the corresponding long one. The result shows one of the limitations of segment-based approach.

In Figure 8, the proposed model detected characters and spatial relationships, such as “in_front_of” and “towards” well. However, the temporal range of the behavioral relationship <child-watch-ball> detected by the proposed model did not extend for long from the beginning of the video to the middle, ending shortly in the middle. This happened due to the following reason. First, the object “ball” was hidden by another object “child” in the middle of the video, disappearing from the video for a while and then reappearing. Therefore, the object tracklet detector of the proposed model could not detect the tracklet of the longer “ball” including the section where the “ball” temporarily disappeared. For this reason, the proposed model only detected a shorter temporal range of the <child-watch-ball> relationship. Current object detection technology and object tracking technology that rely only on visual input cannot solve this problem realistically. Only by applying commonsense reasoning based on the scenes before and after the section where the object disappears from the video will we be able to estimate the position and size of the object during the section of disappearance. Technology that can accurately predict the scene, including the disappeared objects during a specific section of the video based on the scenes before and after the section, is expected to further enhance the ability of the proposed model to comprehend a video. The MAGUS.Gamma model [4] erroneously detected “ball” as “toy” from the beginning of the video. Therefore, the model could not also detect relationships including the object “toy”. The results emphasize the importance of object tracklet detection in video scene graph generation task.

## 5. Conclusions

In this study, a novel deep neural network model, VSGG-Net was proposed for video scene graph generation (VidSGG). We first presented the design issues faced by existing VidSGG models. Our proposed model effectively copes with these issues. A new tracklet pair proposal method was identified. The model performs both low-level visual context reasoning and high-level semantic context reasoning. It applies a class weighting technique that increases the weight of sparse relationships in the classification loss function to improve the detection performance for sparse relationships. The effectiveness of the model was validated through experiments using the benchmark datasets VidOR and VidVRD. Meanwhile, VSGG-Net has some limitations in detecting object tracklets with very short or long lengths, as seen in the example in Figure 7. In addition to the efforts to diversify the size of the sliding windows and increase the number of windows compensating for this problem, it would be necessary to continue looking for new improvements. As seen in Figure 8, VSGG-Net does not accurately detect the tracklet of an object that temporarily disappears from the video, due to it being blocked by other objects in the middle of the video. Therefore, further studies will be needed to expand the current proposed model and generate an accurate scene graph even for a specific section of the video where the object has temporarily disappeared based on the scenes before and after the section.

## Figures and Tables

**Table 1:** Notation summary.

Notation	Description
$G_{c}$	Complete graph
$G_{s}$	Sparse graph
$G_{SG}$	Context graph
tIoU	Temporal intersection over union
$o_{}$	Object
$p_{}$	Object class distribution
$M_{}$	Co-occurrence matrix
$\alpha_{ij}^{}$	Attention on the edge connecting two object nodes $o_{i}$ and $o_{j}$
$C_{}^{i}$	Center coordinate of the bounding box of an object $o_{i}$
$S_{}^{i}$	Size of the bounding box of an object $o_{i}$
$Z^{}$	Information received from neighboring nodes

**Table 2:** Performance metrics.

Performance Metrics	Description
R@1, R@50, R@100	R@K is the recall with Top K results. $recall=\frac{\left|\left\{relevant\;ones\right\}\cap^{\text{​}}\left\{retrieved\;ones\right\}\right|}{\left|\left\{relevant\;ones\right\}\right|}$
P@1, P@5, P@10	P@K is the precision with Top K results. $precision=\frac{\left|\left\{relevant\;ones\right\}\cap^{\text{​}}\left\{retrieved\;ones\right\}\right|}{\left|\left\{retrieved\;ones\right\}\right|}$
mAP	mean Average Precision. The mean of the average precision scores for each query

**Table 3:** Results for tracklet pair proposal using different methods on VidOR validation set. (The average number of tracklets in a video = 986).

Method	# of pairs (↓)	R@1 (↑)	P@5 (↑)
None	958,192	-	0.467
TF	21,560	-	20.78
TF+NS	10,235	70.79	30.98
TF+SS	13,992	97.44	31.19
TF+NS+SS (Ours)	13,965	97.37	31.23

**Table 4:** Results of the CR method of different graph neural networks on VidOR validation set.

Method	Relation Detection			Relation Tagging	
	R@50	R@100	mAP	P@1	P@5
GCN	6.11	7.32	6.24	50.70	47.81
S-GCN	7.66	10.51	8.90	55.39	52.54
T-GCN	7.74	10.85	8.97	56.51	54.35
ST-GNN (Ours)	8.15	11.53	9.80	58.44	54.16

**Table 5:** Results of context reasoning using different levels on VidOR validation set.

Level.	Relation Detection			Relation Tagging	
	R@50	R@100	mAP	P@1	P@5
visual reasoning	7.98	11.48	9.76	58.21	54.09
semantic reasoning	5.63	6.42	5.69	50.90	45.02
visual reasoning + semantic reasoning (Ours)	8.15	11.53	9.80	58.44	54.16

**Table 6:** Comparison of the proposed method with state-of-the-art models on VidOR validation set.

Model	Relation Detection			Relation Tagging	
	R@50	R@100	mAP	P@1	P@5
RELAbuilder [8]	1.58	1.85	1.47	33.05	35.27
MAGUS.Gamma [4]	6.89	8.83	6.56	51.20	40.73
MHA [7]	6.35	8.05	6.59	50.72	41.56
VRD-STGC [9]	8.21	9.90	6.85	48.92	36.78
VSGG-Net (Ours)	8.15	11.53	9.80	58.44	54.16

**Table 7:** Comparison of the proposed method with state-of-the-art models on VidVRD test set.

Model	Relation Detection			Relation Tagging		
	R@50	R@100	mAP	P@1	P@5	P@10
VidVRD [3]	5.54	6.37	8.58	43.00	28.90	20.80
GSTEG [6]	7.05	8.67	9.52	51.50	39.50	28.23
VRD-GCN [5]	8.07	9.33	16.26	57.50	41.00	28.50
MHA [7]	10.38	9.53	19.03	57.50	41.40	29.45
VRD-STGC [9]	11.21	13.69	18.38	60.00	43.10	32.24
VSGG-Net (Ours)	11.30	13.74	18.35	61.36	44.41	33.48

## Data Availability

The datasets used and/or analyzed during the current study are available from the corresponding author upon reasonable request.
